# The Succinate–HIF-1α–NLRP3 Axis in 3-Nitropropionic Acid-Induced Ovarian Dysfunction: A Testable Metabolic–Inflammatory Framework

**DOI:** 10.3390/biology15171557

**Published:** 2026-09-06

**Authors:** Zhenghong Zhang, Defan Wang, Qinghe Lin, Pingting Guo, Zhengchao Wang

**Affiliations:** 1 Provincial Key Laboratory for Developmental Biology and Neurosciences, College of Life Sciences, Fujian Normal University, Fuzhou 350007, China; zhangzh@fjnu.edu.cn (Z.Z.); qinghelin2025@163.com (Q.L.); 2 Fujian Provincial Key Laboratory of Reproductive Health Research, School of Medicine, Xiamen University, Xiamen 361102, China; defanwang@126.com; 3 College of Animal Science, Fujian Agriculture and Forestry University, Fuzhou 350002, China

**Keywords:** 3-nitropropionic acid, succinate, succinate dehydrogenase, HIF-1α, NLRP3 inflammasome, ovarian reserve, granulosa cells, oxidative stress

## Abstract

3-Nitropropionic acid (3-NPA) is an irreversible inhibitor of succinate dehydrogenase (SDH; mitochondrial complex II) that is widely used to model mitochondrial metabolic stress. Direct reproductive studies now show that 3-NPA can increase ovarian oxidative stress, granulosa-cell apoptosis, follicular atresia, alter ovarian reserve, impair oocyte maturation, and reduce fertility in experimental models. However, no ovarian study has yet demonstrated the complete succinate–HIF-1α–NLRP3 cascade proposed here. We therefore propose a testable framework in which SDH inhibition causes succinate accumulation and impaired respiratory electron flux, while redox stress provides an additional signal for HIF-1α stabilization and NLRP3 activation. Succinate-mediated inhibition of prolyl hydroxylases provides a mechanistic route to HIF-1α stabilization under normoxic or near-normoxic conditions, whereas NLRP3 activation may integrate mitochondrial danger signals with inflammatory signaling. Importantly, HIF-1α is not intrinsically pathogenic in the ovary: physiological HIF-1α signaling supports angiogenesis, ovulation, granulosa-cell survival, autophagy, and luteal remodeling, whereas persistent or excessive activation may become maladaptive. We further refine the proposed metabolic–inflammatory threshold as a measurable state in which combined succinate/redox burden and inflammasome activation exceed the adaptive capacity of a follicular unit. The model predicts that time-resolved measurements of succinate, SDH activity, HIF-1α stabilization, NLRP3 activation, and follicular outcomes should reveal ordered relationships that can be tested by pharmacological and genetic intervention. This review distinguishes direct ovarian evidence from cross-system mechanistic evidence and identifies the experiments required to establish causality.

## Simple Summary

3-Nitropropionic acid (3-NPA) is a mitochondrial toxin that inhibits succinate dehydrogenase and can cause reproductive and ovarian injury. Although direct studies have shown that 3-NPA exposure can increase oxidative stress, impair mitochondrial function, promote granulosa-cell apoptosis, disrupt follicular development, and reduce oocyte competence, the molecular pathway connecting these effects remains incompletely understood. In this review, we examine the available evidence and propose that abnormal succinate accumulation may provide a metabolic link between mitochondrial dysfunction, hypoxia-inducible factor-1α (HIF-1α) signaling, and inflammatory pathways involving the NLRP3 inflammasome, a cellular signaling structure that activates inflammatory responses. Importantly, the complete succinate–HIF-1α–NLRP3 pathway has not yet been directly demonstrated in an ovarian 3-NPA model. We therefore distinguish established ovarian findings from evidence derived from other ovarian models and non-ovarian systems. We further propose a metabolic–inflammatory threshold model, in which the balance between metabolic/redox stress, inflammatory activation, and cellular adaptive capacity may influence whether ovarian cells maintain homeostasis or progress toward injury. This framework provides testable hypotheses for future studies and may help clarify how mitochondrial metabolic stress contributes to ovarian dysfunction and related reproductive disorders.

## 1. Introduction

The ovary integrates follicle recruitment, granulosa-cell proliferation and differentiation, steroidogenesis, ovulation, luteal remodeling, and oocyte competence. These processes are governed by endocrine, metabolic, redox, vascular, and immune signals rather than by a single pathway. The size and persistence of the primordial follicle pool are major determinants of reproductive lifespan, while mitochondrial integrity influences oocyte competence and follicular survival [1,2,3].

Mitochondria are particularly important in oocytes, which contain a very high mitochondrial complement and rely on oxidative metabolism for maturation and early embryogenesis. Granulosa-cell metabolism, however, is more heterogeneous than a simple oxidative phosphorylation (OXPHOS)-dominant model implies. Proliferating granulosa cells can exhibit substantial glycolytic activity, whereas mitochondrial oxidative metabolism remains important for steroidogenesis, differentiation, redox control, and cellular adaptation. Thus, a reduction in OXPHOS should not automatically be interpreted as energetic collapse; its consequence depends on developmental stage, substrate availability, and the capacity for glycolytic compensation [3,4,5,6].

Mitochondrial dysfunction and oxidative stress are implicated in ovarian aging, premature ovarian insufficiency (POI), and other reproductive disorders. Recent ovarian studies also show that metabolic perturbations can alter granulosa-cell function and ovarian reserve, supporting an integrated metabolic view of reproductive dysfunction [7,8,9,10,11].

3-NPA is a naturally occurring toxin and an irreversible inhibitor of SDH. In nervous-system models, its toxicity is closely linked to complex II inhibition, succinate accumulation, impaired respiration, and oxidative stress. Importantly, direct reproductive studies are not absent: mouse, goose, vole, and oocyte models have documented ovarian or gamete toxicity. What remains absent is direct evidence that these ovarian phenotypes are causally organized by a succinate–HIF-1α–NLRP3 pathway [12,13,14,15,16,17,18,19,20,21].

The purpose of this review is therefore twofold. First, we synthesize the primary reproductive literature on 3-NPA and explicitly distinguish demonstrated ovarian observations from mechanistic extrapolation. Second, we develop the succinate–HIF-1α–NLRP3 axis as a hypothesis-generating framework rather than an established ovarian mechanism. Particular attention is given to the biochemical feasibility of reactive oxygen species (ROS) generation after irreversible SDH inhibition, the physiological and pathological duality of HIF-1α, the metabolic heterogeneity of granulosa cells, and a measurable definition of the proposed metabolic–inflammatory threshold.

About review methodology and evidence classification, this article is a narrative review with a focused, reproducible literature-mapping component rather than a systematic review or meta-analysis. A final focused PubMed search was conducted on 28 August 2026 using combinations of “3-nitropropionic acid”, “3-NPA”, “ovary”, “ovarian”, “granulosa cell”, “oocyte”, “follicle”, “ovarian reserve”, “premature ovarian insufficiency”, and “embryo”. Mechanistic searches additionally combined 3-NPA or succinate with “succinate dehydrogenase”, “HIF-1α”, “PHD”, “NLRP3”, “TXNIP”, “pyroptosis”, “ferroptosis”, “mitochondrial ROS”, and “SUCNR1”. Primary reproductive studies were prioritized, and recent primary literature was preferentially incorporated. Studies were selected for relevance to ovarian/reproductive toxicity, mitochondrial metabolism, redox signaling, HIF-1α biology, inflammasome signaling, or candidate interventions. No formal PRISMA screening, risk-of-bias scoring, or quantitative meta-analysis was performed. Claims were classified as direct 3-NPA reproductive evidence, ovarian mechanistic evidence not specifically downstream of 3-NPA, or cross-system mechanistic evidence; links not directly supported by these tiers are presented as hypotheses.

## 2. Biochemical Characteristics of 3-NPA

### 2.1. Sources and Toxicological Properties of 3-NPA

**Figure 1:**
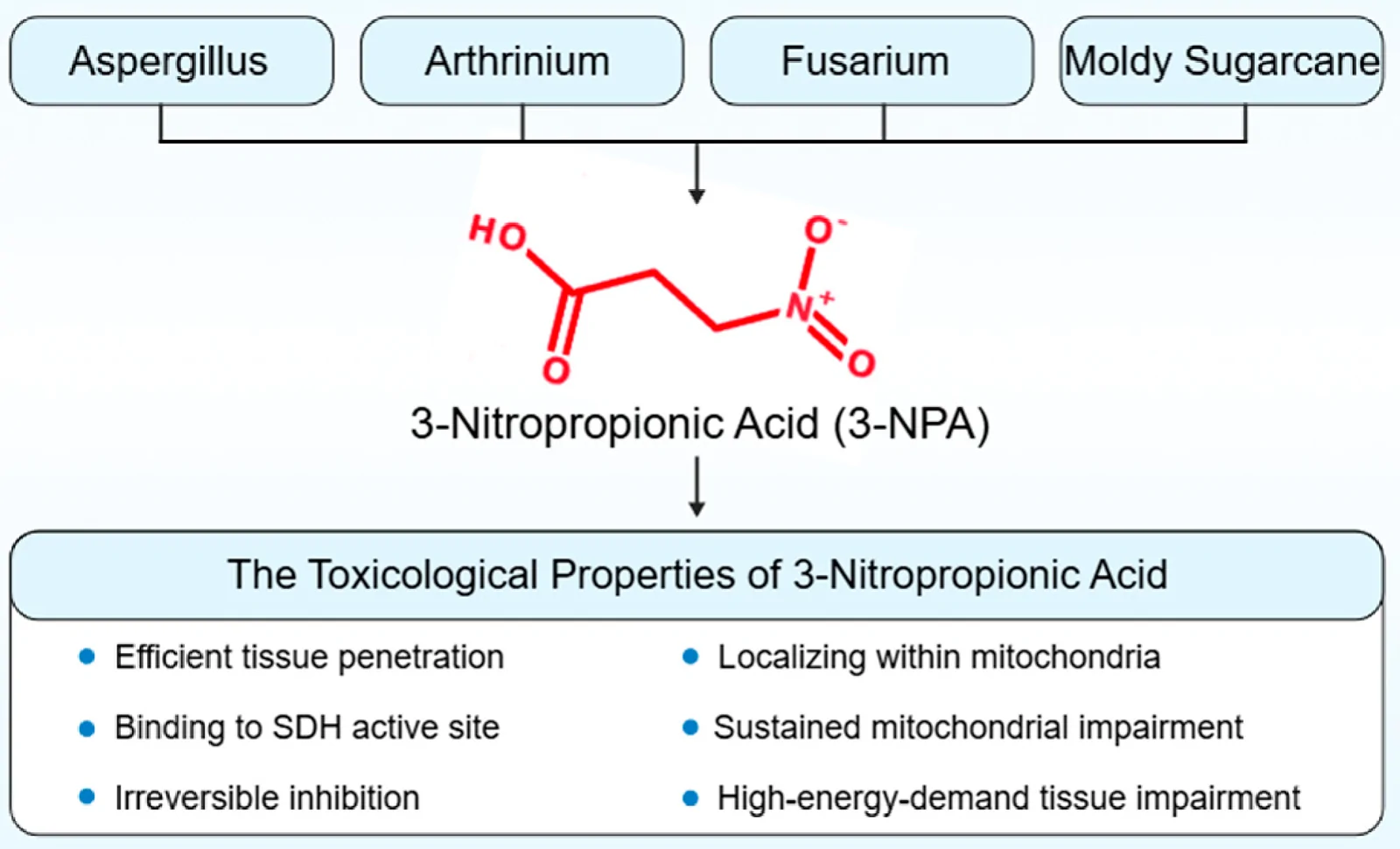
3-NPA as a mitochondrial toxicant and the evidence base for reproductive injury. The figure is retained as a conceptual overview; ovarian phenotypes are supported by primary reproductive studies, whereas downstream pathway links remain hypothesis-driven. Created with BioGDP.com.

3-NPA is produced by several plants and fungi and is associated with poisoning by contaminated food, particularly moldy sugarcane. Its structural relationship to succinate enables interaction with SDH, and covalent modification of the enzyme underlies its irreversible inhibitory behavior [22,23,24] (Figure 1).

The reproductive literature supports biological activity at multiple levels. In female mice, repeated systemic exposure increases ovarian oxidative stress and follicular atresia; in granulosa-cell models it reduces viability and increases apoptosis; in oocytes it disrupts maturation; and in more recent studies it alters ovarian reserve and fertility. These observations establish 3-NPA as an experimental reproductive toxicant, but they do not by themselves identify a common downstream signaling cascade [12,13,14,16,17,18,19,20,21]. 

### 2.2. SDH as the Primary Molecular Target

SDH/complex II links the tricarboxylic acid (TCA) cycle to the respiratory chain. It oxidizes succinate to fumarate while reducing FAD to FADH_2_, and transfers electrons through its iron–sulfur centers to ubiquinone. Structural and biochemical studies establish complex II as a specialized respiratory node whose dysfunction can influence both intermediary metabolism and electron-transfer chemistry [24,25,26].

3-NPA therefore has a distinctive metabolic signature: it blocks succinate oxidation rather than directly inhibiting NADH dehydrogenase. The most defensible upstream event is SDH inhibition, followed by succinate accumulation, reduced complex-II electron entry, altered TCA flux, and secondary changes in cellular redox balance [23,24,26].

### 2.3. Metabolic Consequences of SDH Inhibition

Three consequences are particularly relevant to ovarian toxicity. First, complex-II-dependent respiratory electron flow within the electron transport chain (ETC) is reduced, which can lower respiratory capacity and ATP production when compensatory pathways are insufficient. Second, succinate accumulates because its oxidation to fumarate is blocked. Third, altered respiratory flux can increase ROS generation at mitochondrial redox sites. The magnitude and direction of NADH/NAD^+^ changes are context dependent and should not be described as an obligatory direct consequence of SDH inhibition because complex II is FAD-linked and does not directly produce NADH [26,27,28].

The ROS mechanism also requires correction. Reverse electron transport (RET) at complex I is a well-established ROS-generating mechanism under conditions such as succinate-driven reperfusion, when a large reduced succinate pool feeds electrons into an otherwise competent respiratory chain. Irreversible pharmacological blockade of SDH is biochemically different. Under 3-NPA exposure, it is more appropriate to describe ROS as arising from disturbed complex-II redox chemistry, impaired electron flux, altered ubiquinone redox state, and secondary respiratory imbalance. Complex II itself can generate ROS in both forward and reverse reactions, but the extent to which RET contributes during sustained 3-NPA inhibition in ovarian cells remains untested [27,28,29,30].

### 2.4. Primary Reproductive Evidence for 3-NPA: What Has Actually Been Demonstrated?

A focused PubMed literature search identified a small but expanding body of primary reproductive studies. The evidence is heterogeneous in species, exposure regimen, and endpoint, but it is sufficient to establish direct ovarian and gamete toxicity (Table 1). The studies consistently support oxidative stress, apoptosis, follicular injury, altered ovarian reserve, or impaired oocyte development. In contrast, none of the identified studies simultaneously measured SDH activity/succinate, HIF-1α stabilization, and NLRP3 inflammasome activation in the same ovarian model. This distinction is central to the revised interpretation of the present review.

Table 1 demonstrates that the foundational premise of ovarian 3-NPA toxicity is directly supported, but the proposed axis is not yet directly demonstrated. The most reproducible ovarian signals are oxidative stress, apoptosis, follicular atresia, altered ovarian reserve, and impaired oocyte competence. These findings provide biological plausibility for investigating succinate, HIF-1α, and NLRP3, but they should not be retrospectively relabeled as evidence that those nodes mediate 3-NPA toxicity.

Direct ovarian toxicity should be distinguished from reproductive consequences secondary to systemic 3-NPA toxicity. Direct evidence is strongest when ovarian tissue, isolated granulosa cells, or oocytes are exposed to 3-NPA and the ovarian phenotype is measured directly. In contrast, maternal exposure, offspring phenotypes, and fertility outcomes after systemic dosing may reflect a combination of ovarian and extra-ovarian effects. Because 3-NPA is a potent mitochondrial toxicant with established neurological and systemic toxicity, differences in dose, route, duration, species, and concurrent neurological, metabolic, endocrine, or general toxic effects may influence ovarian endpoints. Accordingly, systemic in vivo studies are interpreted as evidence of reproductive toxicity rather than proof that the ovary is always the primary site of toxicity.

## 3. Ovarian Mitochondria as Targets of 3-NPA

### 3.1. Mitochondrial Dynamics: Direct Evidence Versus Inference

**Figure 2:**
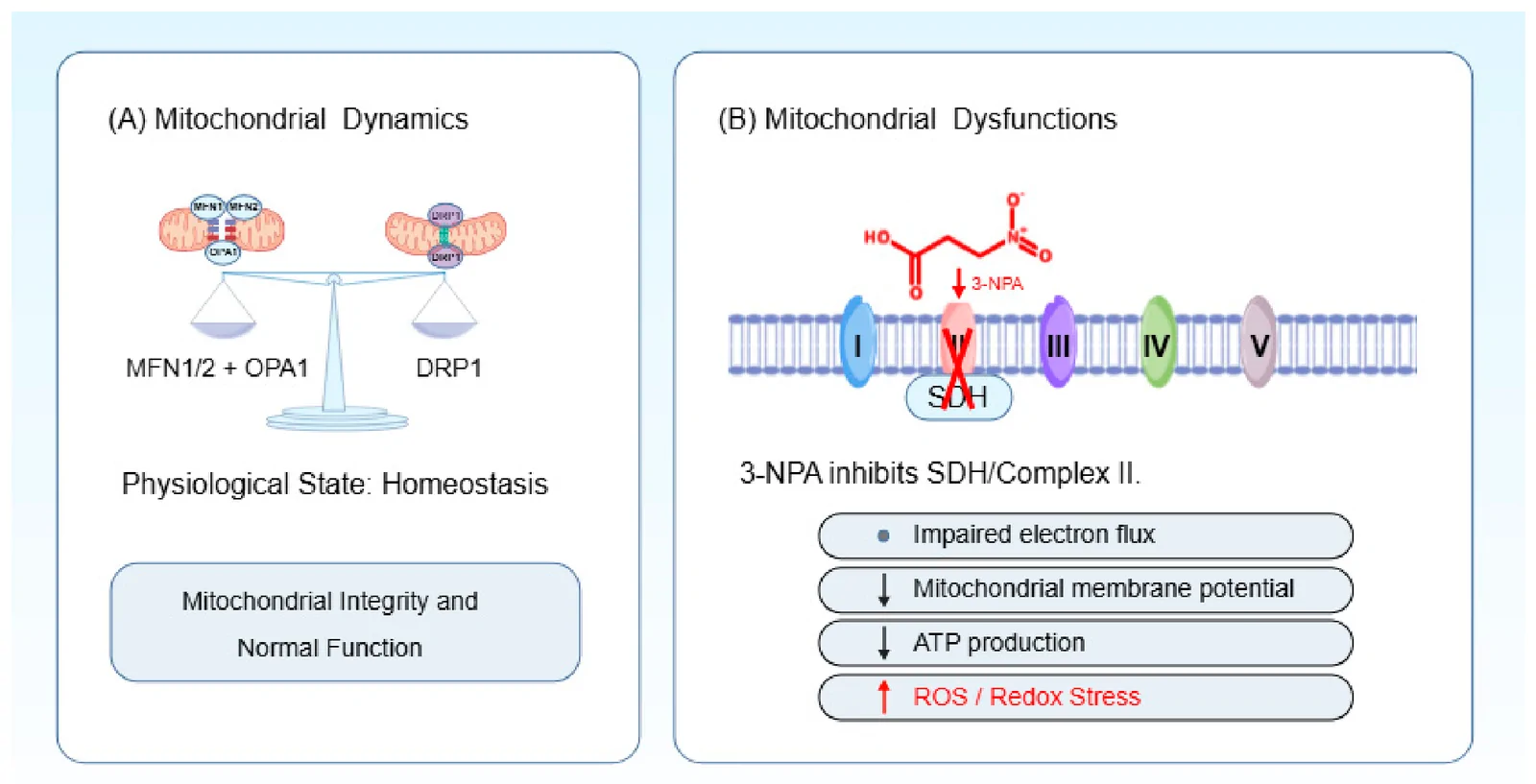
Proposed mitochondrial consequences of 3-NPA in ovarian cells. (**A**) Mitochondrial dynamics, (**B**) Mitochondrial dysfunctions. Direct evidence supports SDH inhibition, oxidative stress, mitochondrial dysfunction and apoptosis. The mitochondrial-dynamics component is shown with increased mitochondrial fission and reduced fusion under 3-NPA-associated metabolic stress. Changes in mitochondrial dynamics and downstream metabolic remodeling remain hypotheses requiring direct validation. ↑ indicates a decrease and ↓ indicates an increase. Created with BioGDP.com.

Direct ovarian studies support mitochondrial and redox disruption after 3-NPA exposure. Reduced SDH activity, altered oxidative-phosphorylation capacity, loss of mitochondrial membrane potential, increased ROS, and impaired antioxidant defenses have been reported in ovarian or granulosa-cell models [12,14,16,17,18,19,20,31] (Figure 2). By contrast, specific remodeling of mitochondrial dynamics, such as DRP1 hyperactivation or coordinated MFN1/2 and OPA1 downregulation, has not been established as a consistent 3-NPA ovarian phenotype. These changes are mechanistically plausible because mitochondrial stress can alter fission/fusion balance, but they should be treated as testable predictions rather than observations [3,5].

### 3.2. Granulosa-Cell Metabolism Is Not Simply OXPHOS Versus Glycolysis

The revised manuscript avoids describing granulosa cells as uniformly OXPHOS-dependent. Proliferating granulosa cells can rely substantially on glycolysis, while mitochondrial metabolism remains essential for steroidogenesis, differentiation, redox regulation, and adaptation. HIF-1α-driven glycolysis may therefore be adaptive in some granulosa-cell states and maladaptive in others. The relevant question is not whether glycolysis increases, but whether the shift is sufficient to maintain ATP demand and biosynthetic functions while preserving redox and mitochondrial homeostasis [5,6,32].

### 3.3. Oxidative Stress and Cell Death

Oxidative stress and apoptosis are the strongest mechanistic findings across the direct reproductive literature. Mouse and goose granulosa-cell models show increased ROS and activation of apoptotic pathways, while more recent studies implicate TRPM2-dependent Ca^2+^ influx, NRF2 signaling, and JNK/ERK responses. Ferroptosis and pyroptosis are biologically relevant forms of granulosa-cell death in other ovarian injury models, but their co-activation by 3-NPA itself remains unproven [12,14,17,18,19,20,33].

Thus, the revised framework distinguishes three evidence layers: (i) direct 3-NPA reproductive toxicity, (ii) ovarian mechanisms demonstrated with other insults, and (iii) cross-system biochemical mechanisms that generate hypotheses for 3-NPA. The proposed succinate–HIF-1α–NLRP3 axis belongs primarily to the third category, with selected nodes supported by independent ovarian literature.

## 4. Succinate Accumulation and HIF-1α-Mediated Pseudohypoxia

### 4.1. Succinate as a Signaling Metabolite

**Figure 3:**
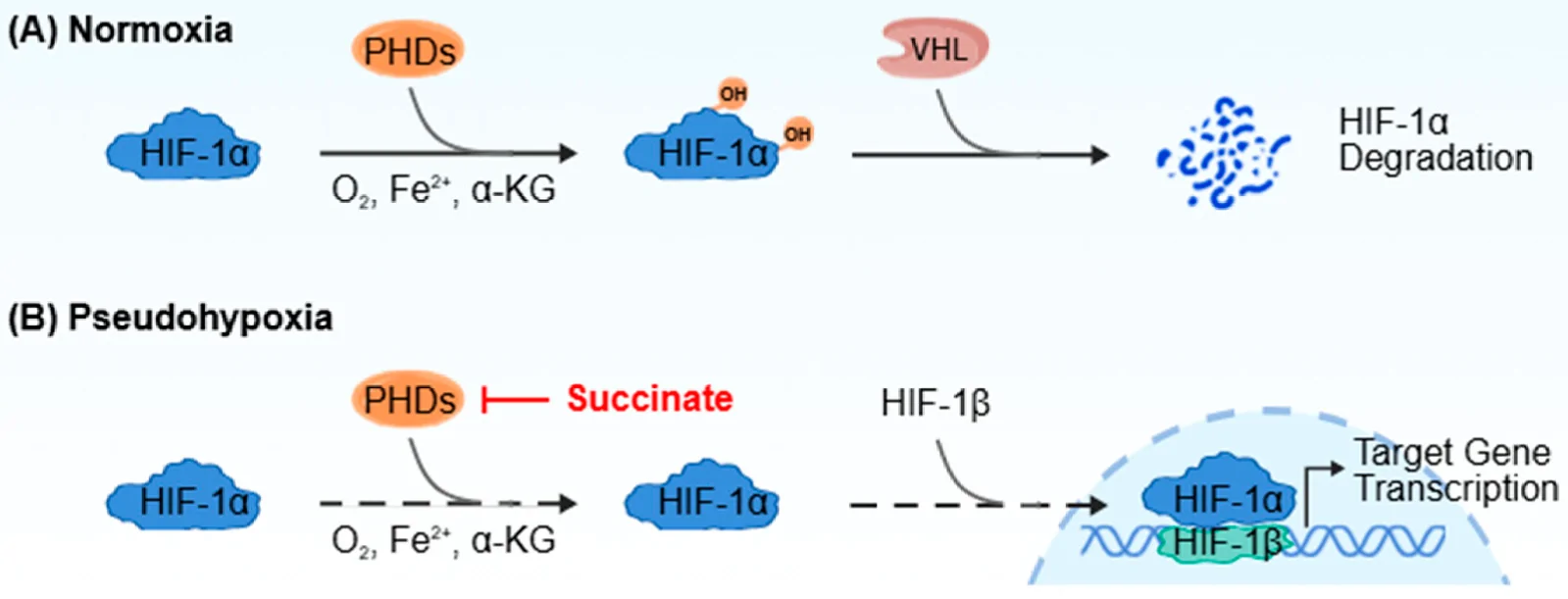
Succinate–PHD–HIF-1α signaling (**A**) and pseudohypoxia (**B**). The figure depicts a mechanistically established biochemical module superimposed on a proposed ovarian context; the dashed boundary marks the proposed 3-NPA-specific integration, and true tissue hypoxia and pseudohypoxia should be experimentally distinguished. Created with BioGDP.com.

Succinate is both a TCA-cycle intermediate and a signaling metabolite. Accumulation of succinate can inhibit α-ketoglutarate-dependent dioxygenases, including prolyl hydroxylases (PHDs), thereby altering HIF stability and other oxygen-sensitive or epigenetic processes. This mechanism is well established in non-ovarian systems [34,35,36] (Figure 3). For 3-NPA-exposed ovaries, however, succinate accumulation should currently be considered a strong biochemical prediction rather than a universally demonstrated ovarian measurement. The most informative experiment would be a time-resolved analysis of intracellular succinate alongside SDH activity and fumarate, before and after 3-NPA exposure. Extracellular succinate signaling through SUCNR1/GPR91 is also plausible, but functional SUCNR1 signaling specifically in granulosa cells after 3-NPA exposure has not been established and should therefore remain explicitly hypothetical.

### 4.2. PHD Inhibition and Pseudohypoxia

Under normoxia, PHD enzymes hydroxylate HIF-1α and facilitate recognition by Von Hippel–Lindau tumor suppressor (VHL). Succinate can inhibit PHD activity and stabilize HIF-1α despite the absence of severe tissue hypoxia. This is appropriately termed pseudohypoxia. Importantly, pseudohypoxia refers to activation of hypoxia-responsive signaling without requiring a fall in tissue oxygen tension; because SDH inhibition can also reduce oxygen consumption, local oxygen availability may even increase in some contexts [36,37,38].

The proposed ovarian sequence is therefore: SDH inhibition → succinate accumulation → reduced PHD activity → delayed HIF-1α degradation. This sequence is biochemically well supported, but its magnitude, timing, and contribution to 3-NPA-induced ovarian injury require direct ovarian measurements.

### 4.3. HIF-1α Is Physiologically Protective as Well as Potentially Maladaptive

HIF-1α should not be characterized as inherently pathological. In the ovary, HIF-1α participates in follicular angiogenesis, ovulation, granulosa-cell survival, autophagy, luteinization, and luteal remodeling. In mice, the FSH–HIF-1α–VEGF pathway is required for ovulation and oocyte health, while granulosa-cell HIF-1α can protect against hypoxia-induced apoptosis and contribute to autophagy [6,32,39,40].

The relevant distinction is therefore between adaptive and maladaptive HIF-1α activity. Transient HIF-1α activation may support survival and tissue remodeling, whereas persistent or excessive activation, especially when coupled to redox stress and inflammatory signaling, could become maladaptive. Accordingly, the metabolic-burden term M in the threshold model refers specifically to persistent or pathological metabolic stress associated with sustained HIF-1α activation, not to physiological HIF-1α pulses required for normal follicular or luteal function. This distinction is essential when interpreting succinate-driven HIF-1α stabilization in a toxicological setting.

### 4.4. Testable Consequences of the Succinate–HIF-1α Module

If succinate is a causal intermediate rather than a bystander, then reducing succinate accumulation or restoring PHD activity should attenuate HIF-1α stabilization without necessarily preventing the initial SDH inhibition. Conversely, direct HIF-1α inhibition should reduce HIF-dependent transcription but should not normalize succinate. These experimentally separable predictions provide a means to distinguish causal order from parallel stress responses.

## 5. NLRP3 Inflammasome Activation and HIF-1α Crosstalk

### 5.1. ROS-Dependent NLRP3 Activation

ROS can promote NLRP3 inflammasome activation through several convergent mechanisms, including disruption of redox-sensitive proteins and mitochondrial stress. The TXNIP–NLRP3 mechanism was initially defined in metabolic and inflammatory systems and should not be treated as a demonstrated 3-NPA granulosa-cell mechanism. In ovarian granulosa cells exposed to other metabolic or inflammatory insults, NLRP3, caspase-1, and GSDMD activation have been directly observed, establishing ovarian competence for this inflammatory cell-death pathway [41,42,43,44]. For 3-NPA, the most defensible formulation is therefore that mitochondrial redox stress may provide a signal capable of activating NLRP3, rather than that 3-NPA has already been shown to activate a TXNIP-dependent NLRP3 pathway in the ovary.

### 5.2. HIF-1α–NLRP3 Crosstalk

**Figure 4:**
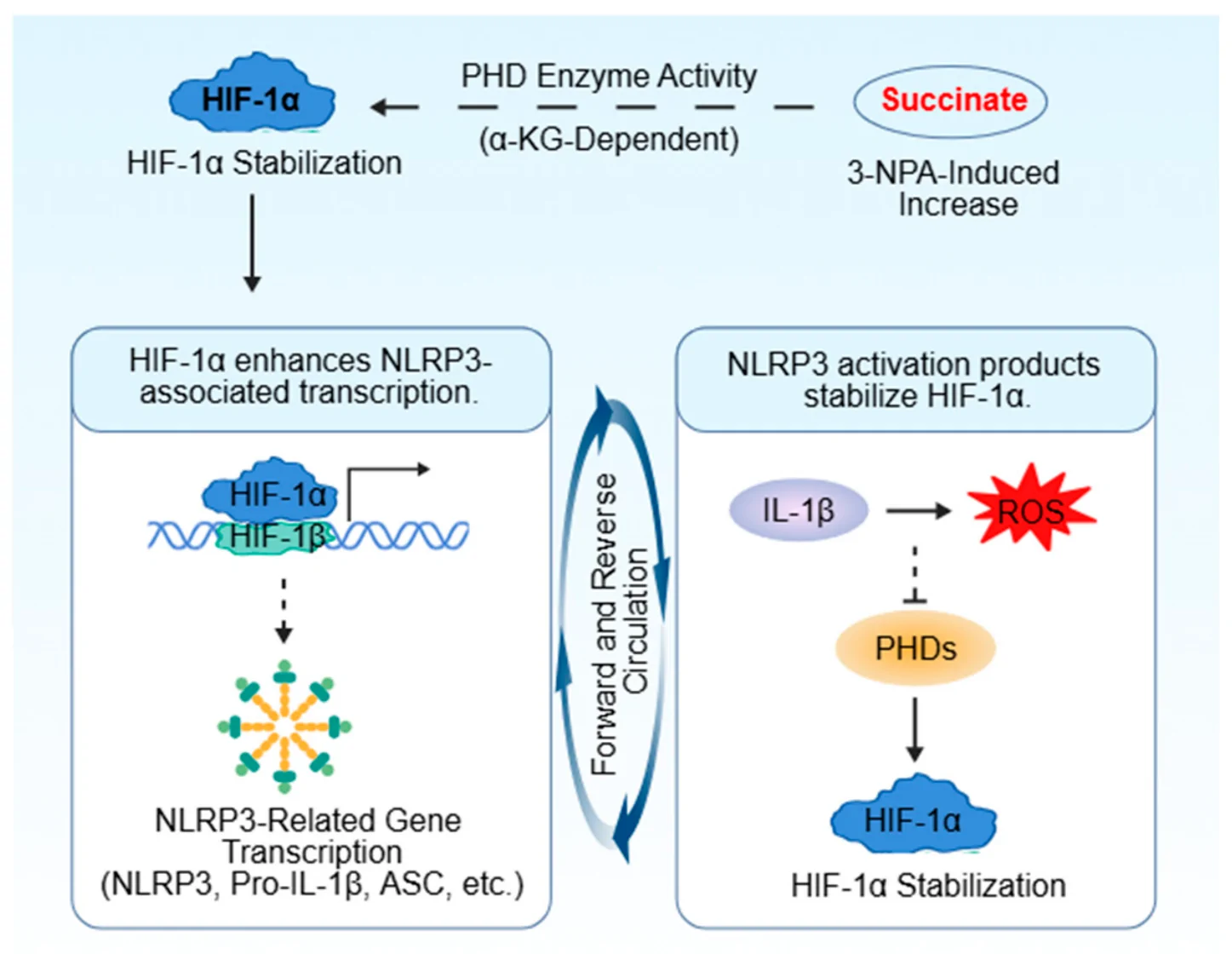
Proposed HIF-1α–NLRP3 crosstalk in 3-NPA-associated ovarian injury. The dashed boundary distinguishes ovarian modules supported by direct or independent evidence from the proposed 3-NPA-specific integration. Created with BioGDP.com.

Cross-system studies support bidirectional coupling between HIF-1α and inflammatory signaling. Succinate can stabilize HIF-1α, and HIF-dependent transcription can promote inflammatory outputs such as IL-1β; conversely, inflammatory signaling can reinforce HIF-1α activity in some contexts [32,35,36]. Ovarian studies independently demonstrate that HIF-1α and NLRP3 are functional regulators of granulosa-cell biology, but direct evidence connecting these two nodes downstream of 3-NPA is lacking [35,36,41,45,46] (Figure 4). The revised model therefore uses a dashed causal boundary between the demonstrated ovarian modules and the proposed 3-NPA-specific integration. This distinction prevents a cross-system mechanism from being presented as if it were already validated in ovarian tissue.

### 5.3. The Metabolic–Inflammatory Threshold: An Operational Definition

We redefine the previously rhetorical “metabolic–inflammatory threshold” as a measurable state variable rather than a fixed biological constant. Let M represent the combined metabolic/redox burden, approximated by normalized succinate accumulation, SDH inhibition, ROS or lipid-peroxidation indices, and mitochondrial membrane-potential loss. Let I represent inflammatory activation, approximated by NLRP3 abundance/assembly, cleaved caspase-1, GSDMD-N, and mature IL-1β/IL-18. Let A represent adaptive capacity, including NRF2 activity, antioxidant capacity, ATP compensation, autophagy, and cell-survival signaling. The threshold hypothesis predicts that follicular injury becomes progressively more likely when M + I exceeds A for a sufficient duration. This formulation generates testable predictions. First, follicular outcomes should correlate more strongly with the integrated time-dependent burden than with any single endpoint. Second, partial reduction in either metabolic stress or inflammasome activation should shift the dose–response curve if the two inputs are coupled. Third, combined intervention should produce greater protection than either intervention alone if the model contains a true synergistic threshold. Fourth, the threshold should be cell-state dependent rather than universal, because granulosa cells, oocytes, theca cells, and stromal/immune cells have different metabolic reserves and signaling networks.

### 5.4. Ovulatory Inflammation Versus Pathological Persistence

Ovulation is accompanied by a tightly regulated inflammatory-like program, and inflammatory mediators participate in follicular rupture and tissue remodeling. The revised manuscript therefore does not equate NLRP3 activation with pathological injury. The proposed threshold concept instead concerns duration, amplitude, and spatial persistence of inflammatory signaling. A transient inflammatory pulse compatible with ovulation should be distinguished experimentally from sustained NLRP3 activation associated with follicular atresia or granulosa-cell death.

## 6. Consequences for Folliculogenesis and Ovarian Reserve

### 6.1. Primordial Follicle Pool

Direct 3-NPA studies support depletion of primordial follicles and development of POI-like phenotypes after repeated exposure. The PI3K–AKT–FOXO3 pathway is a well-established regulator of primordial follicle activation, but the specific sequence 3-NPA → succinate/ROS → PTEN suppression → AKT activation → FOXO3a export has not been demonstrated. It should therefore be treated as a mechanistic prediction rather than an established 3-NPA pathway [1,31].

Inflammation can also influence follicular survival, and ovarian NLRP3 activation is documented in metabolic reproductive disorders. Nevertheless, direct evidence that 3-NPA-induced NLRP3/IL-1β drives abnormal primordial-follicle recruitment is currently lacking. This is an important knowledge gap rather than a settled mechanism [45,46].

### 6.2. Developing Follicles and Granulosa-Cell Function

**Figure 5:**
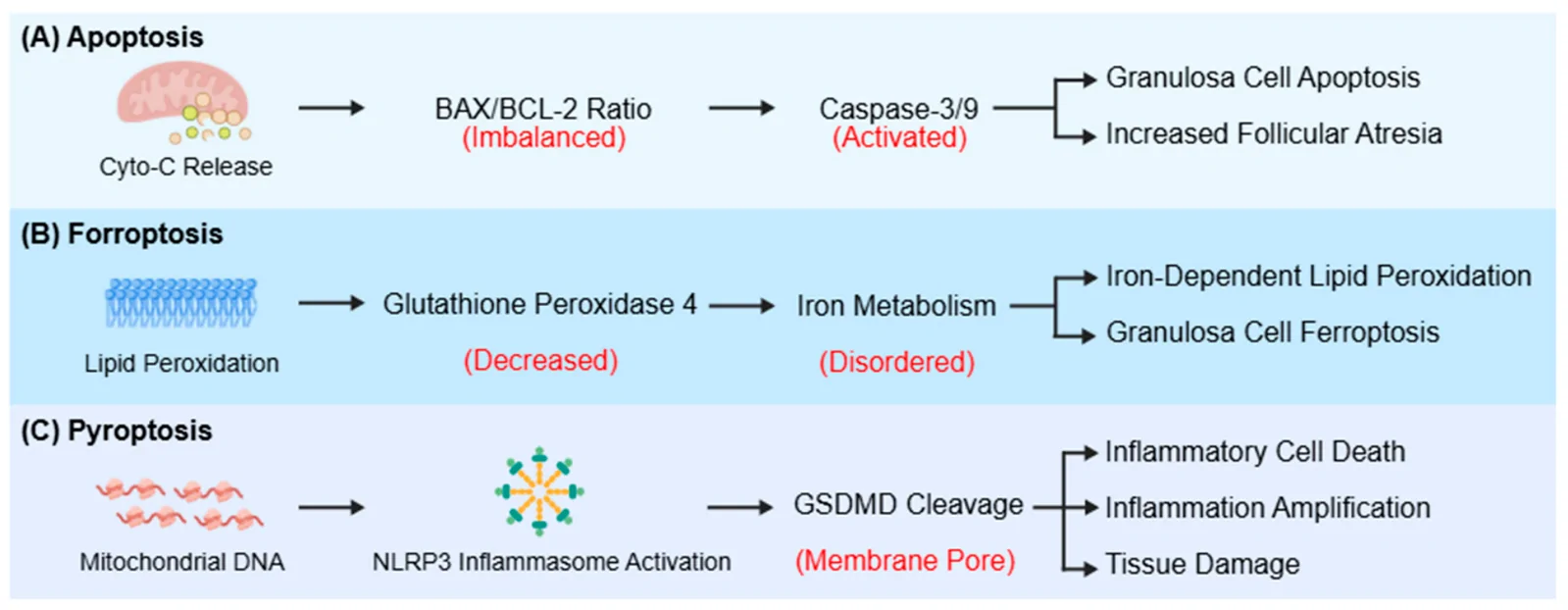
Cell-death pathways potentially contributing to 3-NPA-associated ovarian injury. Apoptosis (**A**) is directly supported by multiple reproductive studies; ferroptosis (**B**) and pyroptosis (**C**) are established ovarian mechanisms in other injury models but remain to be demonstrated as coordinated 3-NPA responses. Created with BioGDP.com.

Direct 3-NPA studies demonstrate impaired granulosa-cell viability/proliferation, increased apoptosis, and reduced follicular development. As an in-principle supporting observation from a different experimental context, IL-1β has been shown to inhibit estrogen formation in cultured rat granulosa cells. This finding demonstrates that inflammatory signaling can affect granulosa-cell steroidogenesis, but it does not establish IL-1β as a mediator of the specific 3-NPA ovarian phenotype, nor does it establish the proposed succinate–HIF-1α–NLRP3 pathway [12,14,17,47] (Figure 5).

### 6.3. Oocyte Quality

3-NPA directly impairs oocyte maturation, including first polar body extrusion, spindle organization, cortical-granule distribution, ATP levels, mitochondrial membrane potential, and redox status. These observations provide direct evidence for gamete-level toxicity. Whether succinate accumulation, HIF-1α stabilization, or NLRP3 signaling contributes to these oocyte phenotypes remains unknown [4,16].

### 6.4. Ovarian Reserve and Endocrine Outcomes

**Figure 6:**
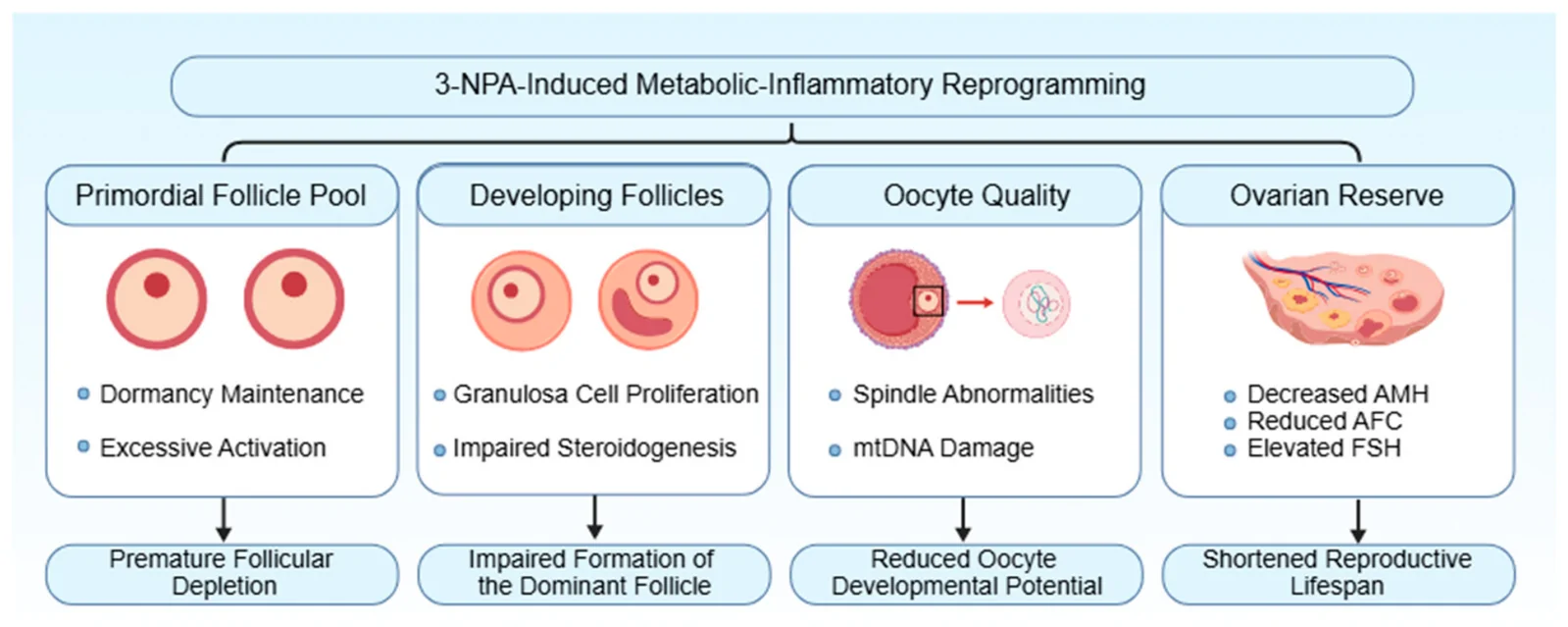
Proposed impact of 3-NPA-associated metabolic and inflammatory stress on folliculogenesis and ovarian reserve. Direct 3-NPA phenotypes are distinguished from proposed signaling links. Created with BioGDP.com.

Repeated 3-NPA exposure can reduce AMH and estradiol, increase FSH, alter the estrous cycle, reduce primordial follicles, increase atresia, and impair fertility in mice. These outcomes support the use of 3-NPA as an oxidative-stress-associated POI model. However, the causal bridge from these phenotypes to the proposed succinate–HIF-1α–NLRP3 axis remains to be established [11,15,18,19,31] (Figure 6).

## 7. Clinical and Disease Relevance: Plausibility, Not Established Causality

### 7.1. Primary Ovarian Insufficiency (POI)

**Figure 7:**
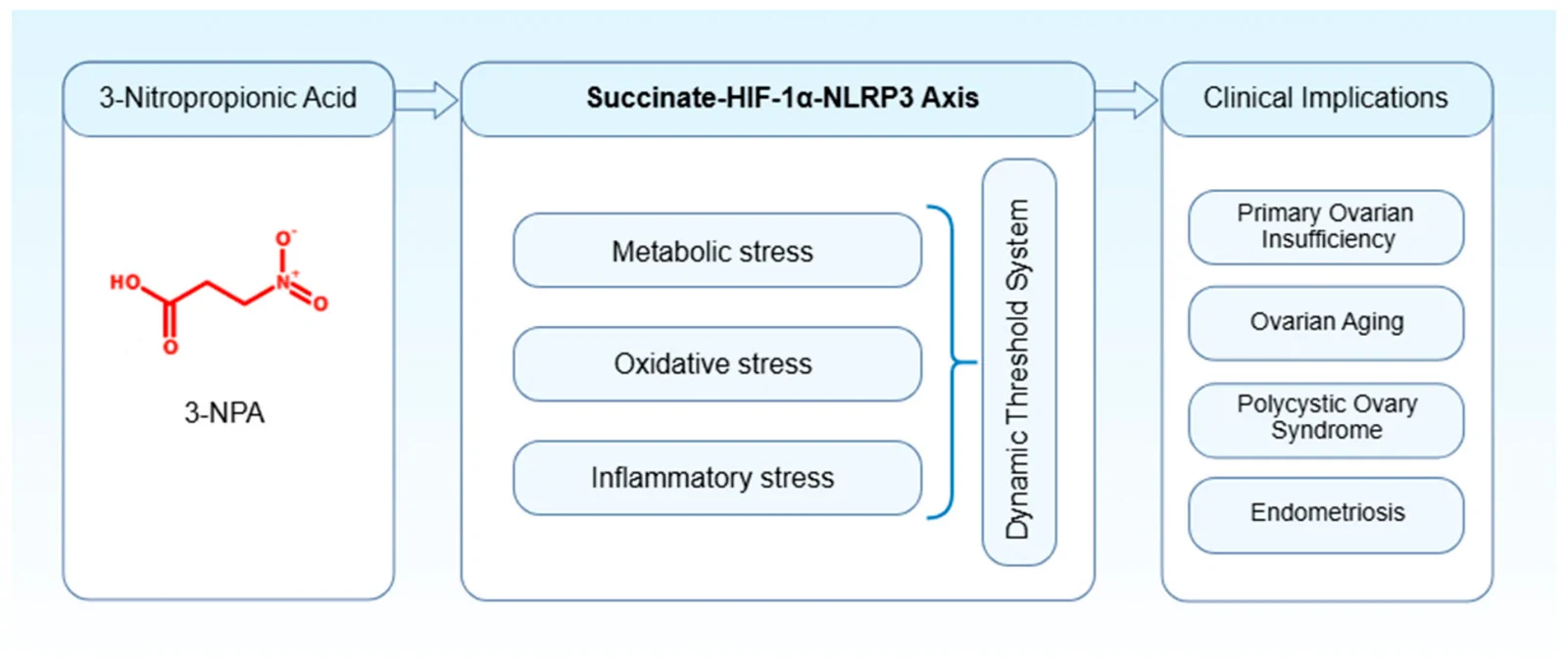
Disease relevance of the proposed succinate–HIF-1α–NLRP3 framework. The figure emphasizes shared biological features rather than implying that the complete axis has been established in POI, ovarian aging, PCOS, or endometriosis. Created with BioGDP.com.

POI is characterized by diminished ovarian reserve and impaired endocrine function. Because 3-NPA can induce POI-like phenotypes in mice and metabolic/redox abnormalities are implicated in human POI, the proposed axis may provide a useful mechanistic hypothesis. However, no evidence currently demonstrates that the succinate–HIF-1α–NLRP3 axis is a causal driver of human POI [11,31,48] (Figure 7).

### 7.2. Ovarian Aging

Ovarian aging is associated with mitochondrial dysfunction, oxidative stress, and chronic low-grade inflammation. These features overlap conceptually with the proposed axis, but overlap is not proof of pathway identity. The strongest value of the model in ovarian aging is therefore as a testable hypothesis linking metabolic stress to inflammatory signaling [3,8,9,49].

### 7.3. Polycystic Ovary Syndrome (PCOS)

NLRP3 activation and granulosa-cell pyroptosis have been demonstrated in PCOS-related models, and metabolic reprogramming is an important feature of PCOS. These independent findings make NLRP3 and HIF-1α biologically relevant to PCOS, but there is no evidence that 3-NPA-induced succinate accumulation explains PCOS pathogenesis. The proposed axis should therefore be presented as a possible mechanistic analogy, not a disease mechanism [45,46,50].

### 7.4. Endometriosis

Endometriosis provides a more defensible disease context for discussing HIF-1α because hypoxia-related signaling and angiogenic remodeling are independently established in the disease. Nevertheless, extrapolating the complete succinate–HIF-1α–NLRP3 sequence from 3-NPA toxicity to endometriosis remains speculative and should be tested in disease-specific models [51].

## 8. Therapeutic Opportunities and Experimental Priorities

### 8.1. Antioxidant and Mitochondrial Protection

**Figure 8:**
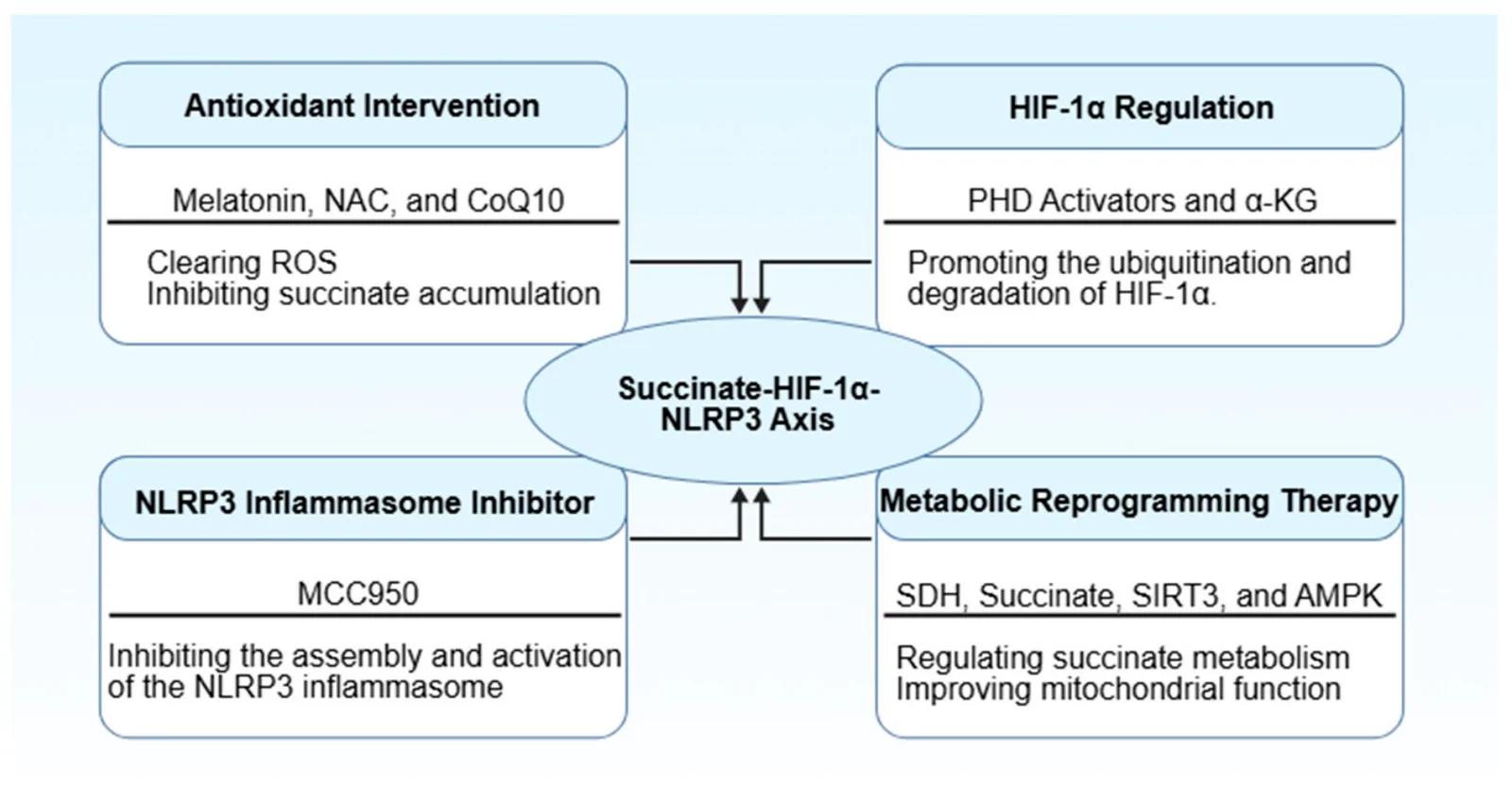
Candidate intervention points for testing the proposed framework. None of the listed strategies should be interpreted as a validated treatment for 3-NPA-induced ovarian injury specifically. Created with BioGDP.com.

Melatonin, N-acetylcysteine, coenzyme Q10, NRF2 activation, and related antioxidant strategies are plausible approaches because oxidative stress is a reproducible component of 3-NPA ovarian injury. However, protection by an antioxidant would not prove the succinate–HIF-1α–NLRP3 pathway; it would establish redox dependence. Direct ovarian 3-NPA studies provide proof-of-principle for several antioxidant or redox-supportive interventions, including proanthocyanidins, tannic acid, silibinin, swertiamarin, selenium, and BCAA supplementation [14,15,17,18,19,22] (Figure 8).

### 8.2. Modulating HIF-1α/PHD Signaling

α-ketoglutarate supplementation or pharmacological modulation of PHD activity could test whether HIF-1α stabilization is downstream of succinate accumulation. The critical experimental requirement is temporal ordering: if PHD restoration reduces HIF-1α stabilization without restoring SDH activity, this would support the proposed metabolite-to-HIF branch. Conversely, excessive HIF-1α inhibition may be harmful because physiological HIF-1α signaling supports ovarian vascular and follicular functions [6,37,39,40].

### 8.3. NLRP3 Inhibition

MCC950 is a well-characterized experimental NLRP3 inhibitor and can be used to test whether inflammasome activation is required for downstream ovarian injury. To the best of our knowledge, MCC950 has not been directly tested as a treatment for 3-NPA-induced ovarian injury. Thus, MCC950 should be described as a mechanistic probe and candidate intervention, not a validated therapy for this model [52].

### 8.4. Metabolic Interventions

SIRT3 is an attractive metabolic node because it regulates mitochondrial homeostasis, antioxidant capacity, and energy metabolism. Yet neither SIRT3/AMPK activation nor direct succinate-lowering strategies have been shown to reverse the full 3-NPA ovarian phenotype. The strongest next step is a factorial design that combines a metabolic intervention with an inflammasome intervention and tests whether combined treatment produces non-additive protection [52,53,54].

## 9. Limitations and Future Perspectives

Several limitations define the current evidence base (Table 2). First, the direct ovarian literature is relatively small and heterogeneous in species, dose, duration, and endpoints. Second, most evidence for succinate–PHD–HIF-1α signaling and HIF-1α–NLRP3 coupling derives from non-ovarian systems. Third, direct measurement of ovarian succinate, HIF-1α hydroxylation/degradation kinetics, NLRP3 assembly, caspase-1 activity, and GSDMD cleavage after 3-NPA is limited or absent. Fourth, 3-NPA is a potent mitochondrial toxicant and may activate parallel pathways, including PARP-1, SIRT1, NRF2, TRPM2, JNK/ERK, autophagy, and glutamine metabolism; the proposed axis may therefore represent one branch of a broader stress response rather than a unique master pathway [14,17,18,19,20,31]. Fifth, pharmacological inhibitors may have off-target effects and cannot by themselves establish pathway order. Genetic approaches, orthogonal metabolic measurements, and rescue experiments are required. Sixth, the dose ranges used in rodents and cultured cells cannot be directly translated into human exposure levels. Finally, HIF-1α and NLRP3 both have physiological functions in ovarian biology, so complete pathway suppression may be neither feasible nor desirable.

The most informative future experiments are therefore: time-resolved ovarian metabolomics for succinate/fumarate and respiratory measurements; direct measurement of HIF-1α hydroxylation, VHL engagement, and transcriptional output; NLRP3 inflammasome assembly and GSDMD cleavage assays in purified granulosa cells; genetic perturbation of SDH, HIF1A, NLRP3, TXNIP, and SUCNR1; cell-type-resolved single-cell or spatial measurements; and factorial intervention studies designed to test the proposed threshold and interaction effects. The recent maternal-exposure study showing an F1 POI-like phenotype further broadens the reproductive evidence base, but its intergenerational design also underscores the need to distinguish ovarian-intrinsic effects from consequences of maternal systemic toxicity and developmental exposure [20].

## 10. Conclusions

Direct reproductive studies establish that 3-NPA is capable of inducing ovarian and gamete toxicity characterized by oxidative stress, granulosa-cell apoptosis, follicular atresia, altered ovarian reserve, impaired oocyte maturation, and reduced fertility. The current evidence does not establish the complete succinate–HIF-1α–NLRP3 pathway in the ovary. We therefore propose this axis as a testable mechanistic framework rather than a proven causal cascade.

The central hypothesis is that irreversible SDH inhibition creates a metabolic state characterized by succinate accumulation and altered mitochondrial redox chemistry; succinate may stabilize HIF-1α through PHD inhibition, while mitochondrial danger signals may promote NLRP3 activation. Physiological HIF-1α and NLRP3 functions must be distinguished from persistent pathological activation. The proposed metabolic–inflammatory threshold is operationally defined as the point at which combined metabolic/redox burden and inflammatory activation exceed adaptive capacity for sufficient duration to alter follicular fate.

The value of this framework is therefore not that it closes the mechanistic question, but that it organizes existing reproductive observations into experimentally separable predictions. Direct measurement and causal perturbation of succinate, HIF-1α, NLRP3, and their temporal relationships will determine whether the proposed axis is a central mechanism, one branch of a broader stress response, or a context-dependent module in 3-NPA-induced ovarian dysfunction.

## Figures and Tables

**Table 1:** Direct evidence for 3-nitropropionic acid (3-NPA)-induced reproductive and ovarian injury across experimental models.

Study/Model	3-NPA Exposure	Direct Reproductive Outcomes	Mechanistic Observations	Evidence Level
Zhang et al., 2014 [10]; ICR mice	6.25, 12.5, 25 mg/kg i.p., 7 d	↓ ovarian weight; ↑ ovarian/GC ROS; ↑ large-follicle atresia; ↓ ovulated oocytes and early embryo development	↑ antioxidant enzymes; ↓ Bcl-2/Bax ratio; granulosa-cell apoptosis	Direct ovarian
Kang et al., 2018 [12]; goose GCs	0.1–20 mM screening; 5 mM, 24 h mechanistic experiments	↓ GC viability; ↑ early apoptosis	↑ ROS; ↑ Bax/p53/cleaved-CASP3; NF-κB and NRF2-related responses	Direct GC
Wei et al., 2019 [11]; female mice	3-NPA exposure; regimen reported in study	↑ atretic and multi-oocyte follicles; ↓ E_2_ and progesterone; ↓ GC proliferation	↓ SDH; altered antioxidant enzymes; PARP-1 activation/cleavage	Direct ovarian
Guo et al., 2022 [13]; mice	40 mg/kg i.p., 3 wk selected POI regimen	↓ primordial follicles, AMH, E2 and fertility; ↑ FSH and atretic follicles	↓ SIRT1; impaired OXPHOS; ↑ MDA; ↓ SOD/GSH-Px	Direct ovarian
Guo et al., 2023 [14]; mice	3-NPA challenge; dose/regimen not consistently reported in the primary article	POI-like ovarian phenotypes; BCAA protection	Ceramide–ROS axis linked to 3-NPA-induced POI	Direct ovarian
Yu et al., 2024 [15]; Brandt’s vole	12.5 mg/kg i.p., 18 d	↓ ovarian coefficient, E_2_/P_4_/AMH and fertility; fibrosis	oxidative stress, autophagy, AKT/mTOR, PTEN and NRF2 changes	Direct ovarian
Han et al., 2024 [16]; mouse oocytes	50–200 μM; in vitro maturation, 12 h	↓ first polar body extrusion; spindle/cortical-granule abnormalities	↓ GSH, mitochondrial membrane potential and ATP; ↑ ROS; apoptosis/autophagy	Direct oocyte
Gai et al., 2025 [17]; mice	12.5 mg/kg; route/duration not specified in the review source	↑ follicular atresia; ovarian histological injury	JNK/ERK activation; KEAP1/NRF2 response; granulosa-cell apoptosis	Direct ovarian
Mo et al., 2025 [18]; ICR mice/GCs	12.5 mg/kg i.p., once daily for 7 d in vivo; 5–20 mM, 48 h in vitro	↓ secondary/preantral/antral follicles and ovulation; GC injury	NRF2/HO-1/SOD1 responses	Direct ovarian/GC
Bucak & Nazıroğlu, 2026 [19]; mouse + KGN GCs	Mouse and KGN models; dose and duration not reported in the review source	GC oxidative injury and follicular atresia	TRPM2/Ca^2+^ influx, ROS, lipid peroxidation and apoptosis	Direct GC
Guo et al., 2026 [20]; C57BL/6 mice	Maternal C57BL/6 mice: 40 mg/kg/day i.p., 21 d	F1 POI-like phenotype, ↓ primordial follicles/AMH, impaired fertility	glutamine-metabolic dysregulation and NF-κB/TNF-α activation	Direct intergenerational *

* For maternal-exposure studies, “intergenerational/systemic” indicates that the ovarian phenotype was assessed in offspring rather than after direct ovarian exposure. ↑ indicates a decrease and ↓ indicates an increase.

**Table 2:** Evidence levels and experimental parameters for testing the proposed metabolic–inflammatory threshold model.

Axis Node	Direct 3-NPA Reproductive Evidence	Cross-System/Ovarian Supporting Evidence	Key Gap	Critical Test
SDH inhibition	Direct: reduced SDH activity reported	Biochemical structure and irreversible inhibition established	Quantitative ovarian SDH–succinate kinetics	SDH activity + succinate/fumarate time course
Succinate	Predicted from SDH blockade; limited direct ovarian quantification	Succinate is an established signaling metabolite.	Ovarian succinate and SUCNR1 function after 3-NPA	LC–MS ± SUCNR1 blockade
HIF-1α	Not directly demonstrated downstream of 3-NPA	Ovarian HIF-1α functions in ovulation, survival and luteal biology	3-NPA-specific HIF stabilization and transcriptional output	HIF-1α hydroxylation, VHL binding, target genes
NLRP3	No direct 3-NPA ovarian causal study identified	NLRP3/pyroptosis demonstrated in metabolic ovarian injury	3-NPA → NLRP3 causality	NLRP3 KO/MCC950 + rescue
HIF-1α ↔ NLRP3	Hypothesis	Bidirectional coupling supported outside 3-NPA ovary	Causal order in granulosa cells	Dual perturbation and temporal profiling
Threshold	Conceptual only	Ovarian cells show variable adaptive capacity.	Operational quantitative boundary	Integrated M + I versus A index; factorial design to estimate interaction term β *

* M + I > A is explicitly defined as the primary additive formulation of the threshold model. If synergistic coupling is presented as a falsifiable extension of the model using an interaction term, M + I + β (M × I) > A, where β > 0 represents positive synergy.

## Data Availability

No new data were created or analyzed in this study. Data sharing is not applicable to this article.

## Abbreviations

The following abbreviations are used in this manuscript:

3-NPA	3-Nitropropionic Acid
α-KG	α-Ketoglutaric Acid
AMH	Anti-Müllerian Hormone
ATP	Adenosine Triphosphate
CAT	Catalase
CoQ10	Coenzyme Q10
CYP19A1	Cytochrome P450, Family 19, Subfamily A, Polypeptide 1
ETC	Electron Transport Chain
FAD	Flavin Adenine Dinucleotide
FOXO3a	Forkhead Box O3a
FSH	Follicle-Stimulating Hormone
GC	Granulosa Cell
GLUT1	Glucose Transporter 1, SLC2A1
GSDMD	Gasdermin D
GSH	Glutathione
LDHA	Lactate Dehydrogenase A
LH	Luteinizing Hormone
HIF-1α	Hypoxia-Inducible Factor-1α
HPO	Hypothalamic-Pituitary-Ovarian Axis
IL-1β	Interleukin-1β
NAC	N-Acetylcysteine
NLRP3	NOD-Like Receptor Family Pyrin Domain-Containing 3
OXPHOS	Oxidative phosphorylation
PHD	Prolyl Hydroxylase
PCOS	Polycystic Ovary Syndrome
PDK1	Pyruvate Dehydrogenase Kinase 1
PI-3K	Phosphatidylinositol-3 Kinase
PKB/Akt	Protein Kinase B
POI	Primary Ovarian Insufficiency
PTEN	Phosphatase and Tensin Homolog
mtROS	Mitochondrial ROS
RET	Reverse Electron Transport
ROS	Reactive Oxygen Species
SDH	Succinate Dehydrogenase, Mitochondrial Complex II
SOD	Superoxide Dismutase
SUCNR1	Succinate Receptor 1
TCA	Tricarboxylic Acid
TfR1	Transferrin Receptor 1
TRX	Thioredoxin
TXNIP	Thioredoxin-Interacting Protein
VEGF	Vascular Endothelial Growth Factor
VHL	Von Hippel–Lindau Tumor Suppressor

## References

[1] Findlay J.K., Hutt K.J., Hickey M., Anderson R.A. (2015). How is the number of primordial follicles in the ovarian reserve established?. Biol. Reprod..

[2] Richards J.S., Pangas S.A. (2010). The ovary: Basic biology and clinical implications. J. Clin. Investig..

[3] May-Panloup P., Boucret L., de la Barca J.M.C., Desquiret-Dumas V., Ferré-L’Hotellier V., Morinière C., Descamps P., Procaccio V., Reynier P. (2016). Ovarian ageing: The role of mitochondria in oocytes and follicles. Hum. Reprod. Update.

[4] Van Blerkom J. (2011). Mitochondrial function in the human oocyte and embryo and their role in developmental competence. Mitochondrion.

[5] Kang M.H., Kim Y.J., Lee J.H. (2023). Mitochondria in reproduction. Clin. Exp. Reprod. Med..

[6] Liang J., Huang F., Hao X., Zhang P., Chen R. (2024). Nicotinamide mononucleotide supplementation rescues mitochondrial and energy metabolism functions and ameliorates inflammatory states in the ovaries of aging mice. MedComm.

[7] Wang L., Tang J., Wang L., Tan F., Song H., Zhou J., Li F. (2021). Oxidative stress in oocyte aging and female reproduction. J. Cell. Physiol..

[8] Zhang X., Zhang L., Xiang W. (2025). The impact of mitochondrial dysfunction on ovarian aging. J. Transl. Med..

[9] Ju W., Zhao Y., Yu Y., Zhao S., Xiang S., Lian F. (2024). Mechanisms of mitochondrial dysfunction in ovarian aging and potential interventions. Front. Endocrinol..

[10] Zhang J.Q., Shen M., Zhu C.C., Yu F.X., Liu Z.Q., Ally N., Sun S.C., Li K., Liu H.L. (2014). 3-Nitropropionic acid induces ovarian oxidative stress and impairs follicle in mouse. PLoS ONE.

[11] Wei Q., Wu G., Xing J., Mao D., Hutz R.J., Shi F. (2019). Roles of poly (ADP-ribose) polymerase 1 activation and cleavage in induction of multi-oocyte ovarian follicles in the mouse by 3-nitropropionic acid. Reprod. Fertil. Dev..

[12] Kang B., Wang X., Xu Q., Wu Y., Si X., Jiang D. (2018). Effect of 3-nitropropionic acid inducing oxidative stress and apoptosis of granulosa cells in geese. Biosci. Rep..

[13] Guo L., Liu X., Chen H., Wang W., Gu C., Li B. (2022). Decrease in ovarian reserve through the inhibition of SIRT1-mediated oxidative phosphorylation. Aging.

[14] Guo X., Zhu Y., Guo L., Qi Y., Liu X., Wang J., Zhang J., Cui L., Shi Y., Wang Q. (2023). BCAA insufficiency leads to premature ovarian insufficiency via ceramide-induced elevation of ROS. EMBO Mol. Med..

[15] Yu M., Fan R., Wang D., Han Y., Dai X., Yang S.M. (2024). Tannic acid alleviates 3-nitropropionic acid-induced ovarian damage in Brandt’s vole (*Lasiopodomys brandtii*). Reprod. Sci..

[16] Han T., Sun Z., Zhang H., Zhao Y., Jiao A., Gao Q. (2024). Ursolic acid alleviates meiotic abnormalities induced by 3-nitropropionic acid in mouse oocytes. Toxicol. Appl. Pharmacol..

[17] Gai Y., Wu W., Wang H., Li Y., Li C., Wang Y., Zhao Y., Hu J., Luan X. (2025). Silibinin attenuates 3-nitropropionic acid-induced ovarian toxicity by alleviating oxidative stress and granulosa cell apoptosis. Reprod. Toxicol..

[18] Mo L., Yang G., Liu D., Zhang H., Dong X., Li F., Huang Z., Zhang D., Xiong Y., Xiong X. (2025). Swertiamarin rescues 3-NPA-induced defective follicular development via modulating the NRF2/HO-1 signaling pathway in granulosa cells. Antioxidants.

[19] Bucak M., Nazıroğlu M. (2026). Selenium protects granulosa cells from 3-nitropropionic acid-induced oxidative toxicity by regulating TRPM2-induced cell death. Reprod. Toxicol..

[20] Guo L., Yu W., Li B., Liu X., Zhang J., Tang W. (2026). Maternal 3-NPA-induced oxidative stress impairs offspring ovarian function via glutamine metabolic dysregulation and NF-κB-mediated inflammation. Reprod. Toxicol..

[21] Zhou X., Ruan H., Dong L., Yu Y., Sun Y., Xiang H., Cao Y., Ding Z. (2025). 3-Nitropropionic acid exposure inhibits embryo development by disrupting mitochondrial function and inducing oxidative stress. Chem. Biol. Interact..

[22] Zhang J.Q., Xing B.S., Zhu C.C., Shen M., Yu F.X., Liu H.L. (2015). Protective effect of proanthocyanidin against oxidative ovarian damage induced by 3-nitropropionic acid in mice. Genet. Mol. Res..

[23] Liu Z.Q., Shen M., Wu W.J., Li B.J., Weng Q.N., Li M., Liu H.L. (2015). Expression of PUMA in follicular granulosa cells regulated by FoxO1 activation during oxidative stress. Reprod. Sci..

[24] Alston T.A., Mela L., Bright H.J. (1977). 3-Nitropropionate, the toxic substance of *Indigofera*, is a suicide inactivator of succinate dehydrogenase. Proc. Natl. Acad. Sci. USA.

[25] Coles C.J., Edmondson D.E., Singer T.P. (1979). Inactivation of succinate dehydrogenase by 3-nitropropionate. J. Biol. Chem..

[26] Sun F., Huo X., Zhai Y., Wang A., Xu J., Su D., Bartlam M., Rao Z. (2005). Crystal structure of mitochondrial respiratory membrane protein complex II. Cell.

[27] Bezawork-Geleta A., Rohlena J., Dong L., Pacak K., Neuzil J. (2017). Mitochondrial complex II: At the crossroads. Trends Biochem. Sci..

[28] Quinlan C.L., Orr A.L., Perevoshchikova I.V., Treberg J.R., Ackrell B.A., Brand M.D. (2012). Mitochondrial complex II can generate reactive oxygen species at high rates in both the forward and reverse reactions. J. Biol. Chem..

[29] Hadrava Vanova K., Kraus M., Neuzil J., Rohlena J. (2020). Mitochondrial complex II and reactive oxygen species in disease and therapy. Redox Rep..

[30] Brand M.D. (2016). Mitochondrial generation of superoxide and hydrogen peroxide as the source of mitochondrial redox signaling. Free Radic. Biol. Med..

[31] Chouchani E.T., Pell V.R., Gaude E., Aksentijević D., Sundier S.Y., Robb E.L., Logan A., Nadtochiy S.M., Ord E.N.J., Smith A.C. (2014). Ischaemic accumulation of succinate controls reperfusion injury through mitochondrial ROS. Nature.

[32] Tannahill G.M., Curtis A.M., Adamik J., Palsson-McDermott E.M., McGettrick A.F., Goel G., Frezza C., Bernard N.J., Kelly B., Foley N.H. (2013). Succinate is an inflammatory signal that induces IL-1β through HIF-1α. Nature.

[33] Mills E., O’Neill L.A. (2014). Succinate: A metabolic signal in inflammation. Trends Cell Biol..

[34] Selak M.A., Armour S.M., MacKenzie E.D., Boulahbel H., Watson D.G., Mansfield K.D., Pan Y., Simon M.C., Thompson C.B., Gottlieb E. (2005). Succinate links TCA cycle dysfunction to oncogenesis by inhibiting HIF-alpha prolyl hydroxylase. Cancer Cell.

[35] Kaelin W.G., Ratcliffe P.J. (2008). Oxygen sensing by metazoans: The central role of the HIF hydroxylase pathway. Mol. Cell.

[36] Semenza G.L. (2012). Hypoxia-inducible factors in physiology and medicine. Cell.

[37] Li C., Liu Z., Li W., Zhang L., Zhou J., Sun M., Zhou J., Yao W., Zhang X., Wang H. (2020). The FSH–HIF-1α–VEGF pathway is critical for ovulation and oocyte health but not necessary for follicular growth in mice. Endocrinology.

[38] Tang Z., Xu R., Zhang Z., Shi C., Zhang Y., Yang H., Lin Q., Liu Y., Lin F., Geng B. (2021). HIF-1α protects granulosa cells from hypoxia-induced apoptosis during follicular development by inducing autophagy. Front. Cell Dev. Biol..

[39] Tang Z., Zhang Z., Lin Q., Xu R., Chen J., Wang Y., Zhang Y., Tang Y., Shi C., Liu Y. (2021). HIF-1α/BNIP3-mediated autophagy contributes to the luteinization of granulosa cells During the formation of corpus luteum. Front. Cell Dev. Biol..

[40] Tang Z., Chen J., Zhang Z., Bi J., Xu R., Lin Q., Wang Z. (2021). HIF-1α activation promotes luteolysis by enhancing ROS levels in the corpus luteum of pseudopregnant rats. Oxidative Med. Cell. Longev..

[41] Wang J., Wu X. (2020). The effects of mitochondrial dysfunction on energy metabolism switch by HIF-1α signalling in granulosa cells of polycystic ovary syndrome. Endokrynol. Pol..

[42] Vander Heiden M.G., Cantley L.C., Thompson C.B. (2009). Understanding the Warburg effect: The metabolic requirements of cell proliferation. Science.

[43] Zhou R., Tardivel A., Thorens B., Choi I., Tschopp J. (2010). Thioredoxin-interacting protein links oxidative stress to inflammasome activation. Nat. Immunol..

[44] Swanson K.V., Deng M., Ting J.P. (2019). The NLRP3 inflammasome: Molecular activation and regulation to therapeutics. Nat. Rev. Immunol..

[45] Shi J., Zhao Y., Wang K., Shi X., Wang Y., Huang H., Zhuang Y., Cai T., Wang F., Shao F. (2015). Cleavage of GSDMD by inflammatory caspases determines pyroptotic cell death. Nature.

[46] Xu R., Zhao H., Qi J., Yao G., He Y., Lu Y., Zhu Q., Wang Y., Ding Y., Zhu Z. (2023). Local glucose elevation activates pyroptosis via NLRP3 inflammasome in ovarian granulosa cells of overweight patients. FASEB J..

[47] Wang B., Shi M., Yu C., Pan H., Shen H., Du Y., Zhang Y., Liu B., Xi D., Sheng J. (2024). NLRP3 inflammasome-dependent pathway is involved in the pathogenesis of polycystic ovary syndrome. Reprod. Sci..

[48] Stuenkel C.A., Gompel A. (2023). Primary ovarian insufficiency. N. Engl. J. Med..

[49] Zeng Y., Wang C., Yang C., Shan X., Meng X.Q., Zhang M. (2024). Unveiling the role of chronic inflammation in ovarian aging: Insights into mechanisms and clinical implications. Hum. Reprod..

[50] Wang Y., Wu J., Zhang G., Shi Y., Meng Y., Lv P., Huang W., Su Y., Zhou Z., Wang B. (2025). Androgens drive SLC1A5-dependent metabolic reprogramming in polycystic ovary syndrome. Nat. Commun..

[51] Giudice L.C. (2010). Clinical practice. Endometriosis. N. Engl. J. Med..

[52] Hirschey M.D., Shimazu T., Goetzman E., Jing E., Schwer B., Lombard D.B., Grueter C.A., Harris C., Biddinger S., Ilkayeva O.R. (2010). SIRT3 regulates mitochondrial fatty-acid oxidation by reversible enzyme deacetylation. Nature.

[53] Coll R.C., Robertson A.A., Chae J.J., Higgins S.C., Muñoz-Planillo R., Inserra M.C., Vetter I., Dungan L.S., Monks B.G., Stutz A. (2015). A small-molecule inhibitor of the NLRP3 inflammasome for the treatment of inflammatory diseases. Nat. Med..

[54] Zhang J., Xiang H., Liu J., Chen Y., He R.R., Liu B. (2020). Mitochondrial Sirtuin 3: New emerging biological function and therapeutic target. Theranostics.

